# Application of heat stress *in situ* demonstrates a protective role of irradiation on photosynthetic performance in alpine plants

**DOI:** 10.1111/pce.12455

**Published:** 2014-11-28

**Authors:** Othmar Buchner, Magdalena STOLL, Matthias Karadar, Ilse Kranner, Gilbert Neuner

**Affiliations:** Institute of Botany, University of Innsbruck6020, Innsbruck, Austria

**Keywords:** *Ranunculus*, *Rhododendron*, *Senecio*, chlorophyll fluorescence, CO_2_-gas exchange, xanthophyll cycle

## Abstract

The impact of sublethal heat on photosynthetic performance, photosynthetic pigments and free radical scavenging activity was examined in three high mountain species, *Rhododendron ferrugineum*, *Senecio incanus* and *Ranunculus glacialis* using controlled *in situ* applications of heat stress, both in darkness and under natural solar irradiation. Heat treatments applied in the dark reversibly reduced photosynthetic performance and the maximum quantum efficiency of photosystem II (F_v_/F_m_), which remained impeded for several days when plants were exposed to natural light conditions subsequently to the heat treatment. In contrast, plants exposed to heat stress under natural irradiation were able to tolerate and recover from heat stress more readily. The critical temperature threshold for chlorophyll fluorescence was higher under illumination (T_c_^′^) than in the dark (T_c_). Heat stress caused a significant de-epoxidation of the xanthophyll cycle pigments both in the light and in the dark conditions. Total free radical scavenging activity was highest when heat stress was applied in the dark. This study demonstrates that, in the European Alps, heat waves can temporarily have a negative impact on photosynthesis and, importantly, that results obtained from experiments performed in darkness and/or on detached plant material may not reliably predict the impact of heat stress under field conditions.

## Introduction

Many alpine plant species have evolved prostrate growth forms that promote the decoupling of plant body temperature from ambient air (Körner & Larcher 1988) in response to the decrease in mean air temperature with increasing elevation. This decoupling allows canopy temperature (Larcher & Wagner 2010) and leaf temperature to become significantly greater than ambient air temperatures (Salisbury & Spomer 1964; Körner & Cochrane 1983). For example, in the cushion plant *Silene acaulis*, a maximum difference of 22 (Neuner *et al*. 2000) and 24.5 K (Gauslaa 1984) between leaf and ambient air temperatures was measured on calm summer days that were characterized by little wind and high solar irradiation. The heat-trapping ability of cushions, rosettes and other prostrate plant growth forms, however, can also be deleterious. Overheating can cause lethal heat limits to be exceeded, resulting in heat-related injury (Gauslaa 1984; Buchner & Neuner 2003; Körner 2003).

Heat can impair CO_2_-gas exchange even before tissue damage becomes apparent (Larcher *et al*. 1973; Bauer *et al*. 1975). The temperature limit for photosynthetic gas exchange in *Senecio incanus* was 3 K below the temperature resulting in visible heat injury to leaf tissues (Larcher & Wagner 1976). Hence, a negative effect of heat on photosynthetic gas exchange can be expected to be more frequent than visible heat damage to leaves. Inactivation of the net photosynthetic rate in *Ranunculus glacialis* occurred at 38 to 39 °C, whereas visible heat injury to leaves only became apparent at temperatures above 45 °C (Larcher & Wagner 1976; Larcher *et al*. 1997).

Studies on the effects of heat stress on photosynthetic gas exchange of alpine plants are rare (e.g. Larcher & Wagner 1976, Larcher *et al*. 1997) and significant information is lacking. For instance, it is not known for how long, and to what extent, photosynthesis remains impaired as a result of heat stress, and how often such events occur in an alpine environment, even though it is well recognized that the photosynthetic apparatus is vulnerable to heat stress (for review see Ducruet *et al*. 2007) and that photosystem II (PSII) is the most susceptible component (see Carpentier 1999).

In alpine environments, the highest temperatures occur during the midday hours in combination with strong solar irradiation. High irradiation intensity, combined with excessive heat, may lead to photoinhibition and photo-oxidative damage resulting from the production of reactive oxygen species (ROS). High mountain plants appear to be highly resistant to photo-oxidative damage, however, different sources of antioxidant protection seem to be prevalent in different species (Streb *et al*. 1997, 2005; Laureau *et al*. 2011). The xanthophyll cycle, in which excess excitation energy is dissipated as heat, also contributes to photoprotection (Bilger & Björkman 1990; Demmig-Adams 2005). The xanthophyll cycle plays an important role in protecting plants from oxidative stress resulting from excess light, as well as drought, heat and other stress factors (Latowski *et al*. 2011). Various studies on xanthophyll cycle pigments, antioxidants such as ascorbic acid, glutathione, tocopherol and antioxidant-related enzymes in alpine plants (e.g. Wildi & Lütz 1996, Streb *et al*. 1997, 2003, 2005; Dongsansuk *et al*. 2005) have indicated that regulating ROS levels is particularly important in high mountain plants.

The effect of strong irradiation, combined with excessive heat, on photosynthesis has been studied to a much lesser extent than the effect of heat exposure under dark conditions. The few reported studies were conducted in the laboratory on isolated spinach chloroplasts (Weis 1982), pea (Havaux *et al*. 1991, 1999) and barley leaves (Havaux & Tardy 1997; Kalituho *et al*. 2003). Interestingly, a protective effect of high irradiation on the photosynthetic performance of plants subjected to heat stress was reported several decades ago (Schreiber & Berry 1977). In contrast, however, a more recent study reported the opposite effect of strong irradiation on rice leaves exposed to heat stress (Yin *et al*. 2010), in which the CO_2_ assimilation rate declined more when heat stress was applied while exposed in the light condition. Therefore, it is not clear if plants tolerate heat better in the light or in the dark condition, and *in situ* data under full natural irradiation are still lacking.

The recent development of an instrument, the ‘heat tolerance testing system’ (HTTS), allows for the application of controlled heat to plants *in situ* under full solar irradiation or in darkness (Buchner *et al*. 2013). This device provides the ability to study the impact of heat treatments on plants in their natural environment, and determine the severity of the heat stress on the impairment of photosynthesis, as well as the dynamics of photosynthetic recovery over a long time period under fully natural conditions. This minimizes potential artefacts produced using detached plant material or artificial environments in growth chambers.

The objectives of the current study were to determine (1) the extent and duration of the impairment of photosynthetic function by measuring parameters such as maximum quantum yield of PSII, maximum quantum efficiency of carbon assimilation, carbon assimilation rate at a defined irradiation intensity and dark respiration rate after *in situ* application of sublethal heat stress in selected alpine plants; (2) the frequency of occurrence of critically high leaf temperatures that impair photosynthesis under field conditions; and (3) the impact of natural solar irradiation during heat application on photosynthesis compared with heat treatments applied in darkness.

We hypothesized that under the current climatic conditions, (1) extended periods of exposure to sublethal heat stress (heat waves) may cause long-term impairment of photosynthesis; (2) the presence of natural solar irradiation during exposure to sublethal heat may help to protect photosynthetic functions; and (3) heat exposure would lead to the alteration of xanthophyll cycle pigments and free radical scavenging activity.

## Material and Methods

### Plant material

Three alpine plant species were selected, representing different growth forms and environmental conditions. *Rhododendron ferrugineum* L. (Ericaceae) is a woody dwarf-shrub, up to 130 cm in height, with evergreen and leathery leaves. It is a typical representative of dwarf-shrub communities in the alpine timberline ecotone (approximately 1600–2100 m.a.s.l.). *S. incanus* L. subsp. *carniolicus* (Willd.) Braun-Blanq. (Asteraceae) is a herbacous species, 5–15 cm high, with leaves that are densely hairy and form rosettes close to the ground. It is often found in dry and stony grasslands, as well as eroded areas in the sub-alpine and alpine zone (approximately 1800–3000 m.a.s.l.). *Ra. glacialis* L. (Ranunculaceae) is also an herbaceous species (height: 5–15 cm) with fleshy leaves, typically found in the subnival and nival zone from 2300 up to >4000 m.a.s.l. It prefers humid and wet sites within scree material and moraines.

### Study sites

Study site 1 (*Rh. ferrugineum*) was located on a north-facing slope at the timberline of Mt. Patscherkofel (1960 m.a.s.l.; 47°12'N/11°27'E), an outpost of the Tuxer Alps, near Innsbruck, Austria. Study site 2 (*S. incanus*) was located within a south-exposed erosion plane slightly beneath the summit of Mt. Patscherkofel (2165 m.a.s.l.; 47°12'N/11°27'E). Study site 3 (*Ra. glacialis*) was located within a slightly inclined, extended stony moraine in the Ötztal Alps near the Timmelsjoch (2560 m.a.s.l.; 46°54'N/11°09'E).

### Micrometeorology

Micrometeorological data were recorded at 1 min intervals by data loggers (CR10X, CR1000, Campbell Scientific, Loughborough, UK) at each study site. Photosynthetic photon flux density (PPFD) was measured by cosine-corrected quantum sensors (SKP 215, Skye Instruments Ltd., Llandrindod Wells, UK). Thermocouple sensors (Type T; GG-Ti-28, Omega Engineering Inc., Stamford, CT, USA) were placed 2 m above the ground and shielded against direct sunlight for determination of air temperature. Small thermocouple sensors (Type T; TT-Ti-36, Omega Engineering Inc.; solder junction diameter: 0.3 mm) were used to measure leaf temperature and were placed on the lower leaf surface with special leaf clamps to allow unrestricted solar irradiation and leaf transpiration (see Buchner *et al*. 2013).

### Exposure to heat stress

**Figure 1:**
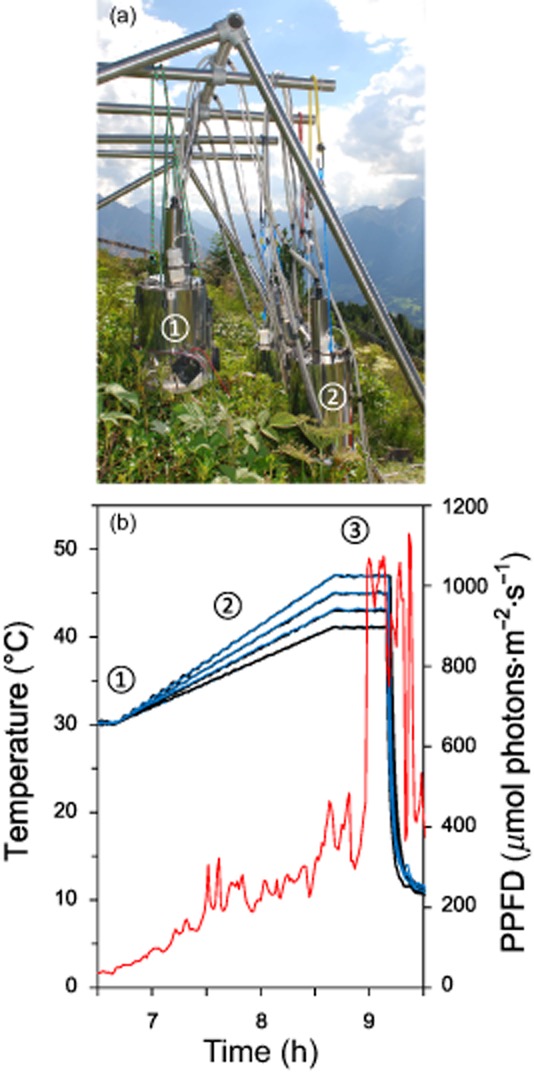
Controlled application of heat to whole twigs of *Rhododendron ferrugineum*. (a) The heat tolerance testing system (HTTS) was used to apply controlled heat to plants *in situ* under full solar irradiation or in darkness on Mt. Patscherkofel (1960 m.a.s.l.). The exposure chambers of the HTTS were operated in the light mode (1) and in the dark mode (2). (b) Examples of temperatures recorded during the time course of the controlled heat treatment in the exposure chambers. Three different phases can be distinguished: (1) stabilization; (2) heating; and (3) exposure. Solid lines, leaf temperature inside the exposure chambers (black, dark mode; blue, light mode); red line, solar irradiation (PPFD) during the heat treatment.

The HTTS was used to expose selected plants to defined levels of heat stress *in situ* in the field in the presence of full natural solar irradiation (light mode) or in darkness (dark mode) (Fig. 1a). The device and method are described in detail in Buchner *et al*. (2013). The system consists of a software-controlled (based on LabView 2012, National Instruments, Austin, TX, USA) central supply unit in which up to eight exposure chambers can be connected. Each exposure chamber is made of cylindrical highly transparent Plexiglas (XT 29070, Röhm, Darmstadt, Germany) into which the samples are inserted. When the exposure chambers are operated in the light mode, the ambient solar irradiation may pass freely through the Plexiglas illuminating the plant samples inside. But it is also possible to keep the plant samples in darkness during the heat treatment (dark mode) which is realized by putting special steel tubes over the Plexiglas window of the exposure chambers. Four leaf temperatures are recorded continuously in each chamber and mean leaf temperature is automatically controlled by the system. In order to ensure homogenous leaf temperatures, relative humidity within each exposure chamber can be increased to create water vapour-saturated air, thereby restricting leaf cooling by transpiration. In addition, 12 small fans ensure sufficient convection inside the chamber to minimize temperature fluctuations of leaves exposed to strong solar irradiation.

The heat treatment of plants always started before sunrise and was divided into three phases (Table 1, Fig. 1b): (1) stabilization phase – after plants were inserted into the exposure chambers, mean leaf temperature was held at a start temperature until the temperature within the exposure chambers stabilized. This phase lasted approximately 15–30 min depending on the environmental conditions; (2) heating phase – plant temperatures were increased over a 2 h period using a constant warming rate until designated target temperatures were reached. The warming rates were similar to natural leaf heating rates measured in natural stands of alpine plants (Neuner & Buchner 2012); and (3) exposure phase – subsequent to the heating phase, plants within the chambers were exposed to a predetermined target temperature for 30 min, a time span that is commonly used for the determination of heat tolerance (Kreeb 1990). The exposure chambers were then opened and removed. The duration (2.5 h) of the entire heat treatment was similar for all of the defined target temperatures. Plants used in the study were exposed to each specific heating regime both in the light mode (abbreviated ‘46L’) and also in the dark mode (‘46D’). This was done in order to study the effect of the presence or absence of natural solar irradiation on photosynthetic gas exchange at each of the selected temperatures (e.g. 46 °C).

**Table 1:** Parameters for the controlled *in situ* heat treatments using the heat tolerance testing system (HTTS)

Species	Date	T_start_ [°C]	WR [K·h^−1^]	T_exp_ [°C]	T_c_ [°C]	LT_5_ [°C]	T [°C]	PPFD
*Rhododendron ferrugineum*	9 August 2012	30	6.5	43	44	46	11	261
*Rh. ferrugineum*	9 August 2012	30	7.5	45	44	46	11	261
*Senecio incanus*	(10) 11 July 2013	30	7	44	45	47	(17) 19	(732) 1047
*S. incanus*	(10) 11 July 2013	30	8	46	45	47	(17) 19	(732) 1047
*Ranunculus. glacialis*	(10) 11 August 2013	25	8	41	43	44	(5) 8	(1351) 1501
*Ra. glacialis*	(10) 11 August 2013	25	9	43	43	44	(5) 8	(1351) 1501

Leaves were treated at each exposure temperature (T_exp_) in the light mode and in the dark mode. T_start_, starting temperature of the heat treatment, WR, warming rate, T_exp_, exposure temperature, T_c_, critical temperature that causes an increase in basic fluorescence, LT_5_, temperature at which first visible heat injury to the leaf blade (5% of total leaf area) occurs. T and PPFD: mean values of leaf temperature and photosynthetic photon flux density [*μ*mol photons·m^−2^ s^−1^] determined for untreated control plants outside the HTTS. Values outside brackets refer to the heat treatment applied to determine the effects on photosynthetic gas exchange, values in brackets (date, T, PPFD) refer to the determination of the effects on free radical scavenging and xanthophyll cycle activities.

### Selection of high temperatures

Two different exposure temperatures, within 2 K of each other, were selected for each species that would induce a severe but sublethal heat stress. The selected temperatures were 3 and 1 K below the temperature that induced the first evidence of heat injury to the leaves (LT_5_), and as close to the critical high temperature threshold of PSII (T_c_) as possible. Prior to the heat exposure, an LT_5_ and T_c_ under dark conditions were calculated to determine the exposure temperatures for each species. Leaf heat tolerance (LT_5_) was determined as described by Buchner & Neuner (2001). Leaf samples (10 samples per temperature) were fixed to overhead transparencies with adhesive tape (3M™ Transpore™, 3M Österreich GmbH, Perchtoldsdorf, Austria). The transparencies were then mounted inside a series of small exposure chambers, which had been preheated to a range of incrementally increasing temperatures. After exposure (30 min; Kreeb 1990), samples were immediately removed and placed into plastic bags, and kept at high humidity under moderate illumination for 20 h, until heat damage to the leaf blade could be assessed by determining the relative proportion of the damaged leaf area to the total leaf area using graphical analysis software (Optimas 6.5, Optimas Corp., Seattle, WA, USA) (Buchner *et al*. 2013). The temperature threshold, defined as the temperature inducing damage to 5% of the total leaf area (LT_5_), was estimated from this data and used to select the target exposure chamber temperatures for each of the species examined.

### Determination of the critical high temperature threshold of F_0_ in darkness and F_s_ at different levels of irradiation

For *S. incanus* and *Ra. glacialis*, the critical high temperature threshold of PSII in darkness (T_c_) was determined *in situ* using the laboratory-based T-F_0_ technique (Schreiber & Berry 1977; Braun *et al*. 2002). Leaves were inserted into the fully darkened standard cuvette of a gas exchange-fluorescence system GFS-3000 (Walz, Effeltrich, Germany) with the temperature range extended up to +60 °C. Leaf surfaces were irradiated with a low pulse-modulated measuring light (470 nm) from light emitting diodes (LED array PAM fluorometer 3055-FL, Walz) that was fixed to the cuvette. Basic fluorescence F_0_ was measured at intervals of 1 s, while leaf temperature was progressively increased at a rate of 1 K min^−1^ up to +55 °C. T_c_ was defined as the temperature at which F_0_ exhibited a distinct increase (Neuner & Pramsohler 2006), indicating the onset of the inactivation of PSII (Larcher *et al*. 1997).

This procedure was also conducted at different levels (10, 30, 100, 200, 650, 1000, 1500, 1650, 1800 *μ*mol photons·m^−2^ s^−1^) of irradiation in order to determine the critical high temperature threshold T_c_′ of F_s_ as a function of irradiation intensity. Leaves were irradiated with actinic light emitted from 90% red (640 nm) and 10% blue (470 nm) LEDs, and steady-state fluorescence F_s_ was measured at intervals of 1 s. The critical temperature (T_c_^′^) was defined as the temperature at which F_s_ showed a sharp increase under the respective level of irradiation.

### Determination of the potential efficiency of PSII

Chlorophyll fluorescence is commonly used for monitoring photosynthetic performance (reviewed by Baker 2008) and has been widely reported to be sensitive to heat stress (e.g. Bilger *et al*. 1984, Weis & Berry 1988, Weng & Lai 2005). Therefore, the maximum quantum yield of PSII (F_v_/F_m_) can be used as a simple indicator of heat stress (Willits & Peet 2001) and for assessing the temperature causing permanent tissue damage (Krause *et al*. 2010). F_v_/F_m_ was determined *in situ* in dark adapted leaves (30 min) using a portable chlorophyll fluorometer (PEA MK2, Hansatech Instruments Ltd., Norfolk, UK) before and repeatedly after controlled *in situ* heat exposure of whole plants in darkness and under natural solar irradiation.

### *In situ* photosynthetic light response curves

The aftereffects of heat stress on photosynthetic gas exchange were assessed using parameters derived from photosynthetic light response curves: (1) dark respiration rate R_d_ (*μ*mol CO_2_ m^−2^ s^−1^), which describes the rate of CO_2_ released by a certain leaf area in darkness; (2) A_2000_, the assimilation rate at high irradiation (PPFD = 2000 *μ*mol photons·m^−2^ s^−1^); (3) Φ, the maximum quantum efficiency defined as the positive linear slope of the curve (*μ*mol CO_2_ mmol^−1^ photons); and (4) the diffusion conductivity for water G_H2O_ (mmol m^−2^ s^−1^), which provides information on stomatal opening or closure. (1) and (3) were calculated by fitting a linear function to the linear part of the light response curve under weak irradiation (0 to 50 *μ*mol photons·m^−2^ s^−1^). (2) and (4) were extrapolated from the gas exchange data by fitting a linear or a polynomial function (Eqn. 1) to the data using NI DIAdem software (2012, National Instruments, Austin, TX, USA).


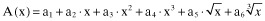
(1)

Measurements were conducted *in situ* at 380 ppm CO_2_ and 10 000 ppm H_2_O at a flow rate of 750 *μ*mol s^−1^. Leaf temperature was held at 20 °C and PPFD was incrementally increased (0, 5, 10, 15, 50, 100, 150, 500, 1000, 1500 and 1800 *μ*mol photons·m^−2^ s^−1^). Each step lasted for 3 min and gas exchange measurements were taken at 1 min intervals.

The low water vapour content of the measuring gas [e.g. 10 000 ppm corresponds to 32% relative humidity (Rh) at an ambient temperature 20 °C and air pressure 750 kPa] was essential, when the ambient air temperature was low, to avoid water condensation inside the measurement cuvette when opening it to insert new leaf samples. Individual samples were always taken at the same time of day in order to minimize diurnal effects on stomatal conductance measurements. All measurements were taken *in situ* on healthy leaves 1 d before conducting the controlled heat exposure experiments on previously untreated leaves and at several time intervals (1, 2, 6, 13 d) after the heat treatment.

### Radical scavenging activity and pigment analysis

#### Heat treatment and sampling

The *in situ* heat treatment of plants used to obtain samples for biochemical analysis followed the same protocol as described earlier for the gas exchange measurements and was conducted 1 d prior to the gas exchange studies. In order to obtain sufficient samples for biochemical analysis from each species, only one sublethal exposure temperature (*S. incanus*: 44 °C; *Ra. glacialis*: 41 °C; *Rh. ferrugineum* was not investigated) was used. Four exposure chambers were operated in dark mode or in light mode in parallel. Randomly selected leaf samples were placed into small paper bags and frozen in liquid nitrogen immediately after each heat treatment. This procedure was conducted in the field under low irradiation (PPFD <50 *μ*mol photons m^−1^ s^−1^) using a black felt blanket until the samples were taken in order to protect plants that were exposed in the dark mode from strong light after the experiment. Samples were transferred within 2 d from liquid nitrogen and stored in a −80 °C freezer for several weeks. Samples were then freeze-dried (Lyovac GT 2, Leybold-Heraeus, Köln, Germany) for at least 3 d and ground to a fine powder using a TissueLyser II (Qiagen, Düsseldorf, Germany) at 1900 rpm for at least 3 min. The powder was transferred into Eppendorf tubes (1.5 mL) and stored at −80 °C until biochemical analyses were conducted.

#### Determination of total free radical scavenging activity

The total free radical scavenging activity was determined using 2,2-diphenyl-1-picrylhydrazyl (DPPH) based on the method described by Brand-Williams *et al*. (1995) and Fukumoto & Mazza (2000). Five milligram of freeze-dried leaf powder was transferred to light-protected (brown) Eppendorf tubes (1.5 mL), suspended in 1 mL methanol (100%) and shaken at 4 °C for 16 h. The samples were then centrifuged at 13 000 *g* at 4 °C for 4 min and the supernatant from each sample was transferred into new Eppendorf tubes and kept on ice for a maximum of 30 min. Subsequently, 22 *μ*L of the supernatant were placed into each well of a 96-well microtitration plate (Bio-Rad, Hercules, CA, USA), 200 *μ*L DPPH solution was added (150 *μ*M DPPH in 80% v/v ethanol) and the plate was kept in the dark. The decrease in absorbance at 520 nm was measured after 5, 10, 15, 20, 25, 30, 35, 60 and 90 min using a plate reader (Multiskan EX, Thermo Fisher, Waltham, MA, USA). The stable free radical ‘Trolox’ (6-hydroxy-2,5,7,8-tetramethylchroman-2-carboxylic) was used to construct a calibration curve (500, 400, 300, 200, 100 and 50 *μ*M Trolox in 100% v/v methanol) and total free radical scavenging activity was expressed as Trolox equivalents (TE), normalized to dry mass.

#### Pigment analysis

Pigments were analysed by high-performance liquid chromatography (HPLC) using a protocol that was slightly amended from that described by Pfeifhofer *et al*. (2002). Fifty milligram of freeze-dried leaf powder was transferred into brown Eppendorf tubes (1.5 mL) together with a spatula tip of CaCO_3_ and suspended in 500 *μ*L dimethylformamide (DMF). After vortexing for 30 s (Ika-Vortex Geneus 3, IKA, Staufen, Germany), the samples were maintained at −21 °C for at least 12 h and then centrifuged (20 000 *g*) at 4 °C. The supernatant from each sample was decanted into brown Eppendorf tubes and stored at –21 °C. The pellet was resuspended in 500 *μ*L DMF and kept at −21 °C for at least 12 h. The procedure was repeated two times using 250 *μ*L for *Ra. glacialis* and three times for *S. incanus*. Subsequently, 500 *μ*L of the combined supernatant from each sample was mixed with 250 *μ*L 50% v/v methanol and centrifuged (20 000 *g*) at 4 °C for 20 min. The resulting supernatant was used for HPLC analysis (Agilent Technologies, Santa Clara, CA, USA). Pigments were separated on a LiChroSpher C18 (Phenomenex Inc., Torrance, CA, USA) column at a flow rate of 1 mL min^−1^. Pigments were identified by retention time and absorption spectra using a diode array detector and quantified using a calibration curve of external standards (chlorophyll *a*: Sigma Aldrich, St. Louis, MO, USA; antheraxanthin and violaxanthin: DHI, Hørsholm, Denmark; zeaxanthin and lutein: Carl Roth, Karlsruhe, Germany; ß-carotene: Calbiochem, Darmstadt, Germany). Neoxanthin and chlorophyll *b* were collected with an Agilent 1200 Series fraction collector (Waldbronn, Germany) and concentrations were calculated using absorption coefficients as described by Pfeifhofer *et al*. (2002) before being used as external standards. Five biological replicates were analysed for each treatment in each experiment.

### Statistics

Differences between mean values were tested either by analysis of variance (anova) in combination with Duncan's multiple range test (*P* < 0.05) or by the *t*-test (*P* < 0.05) and were conducted using SPSS (IBM SPSS-Statistics 21, New York, NY, USA).

## Results

### Micrometeorology

#### *Rh. ferrugineum* (summer 2012)

**Figure 2:**
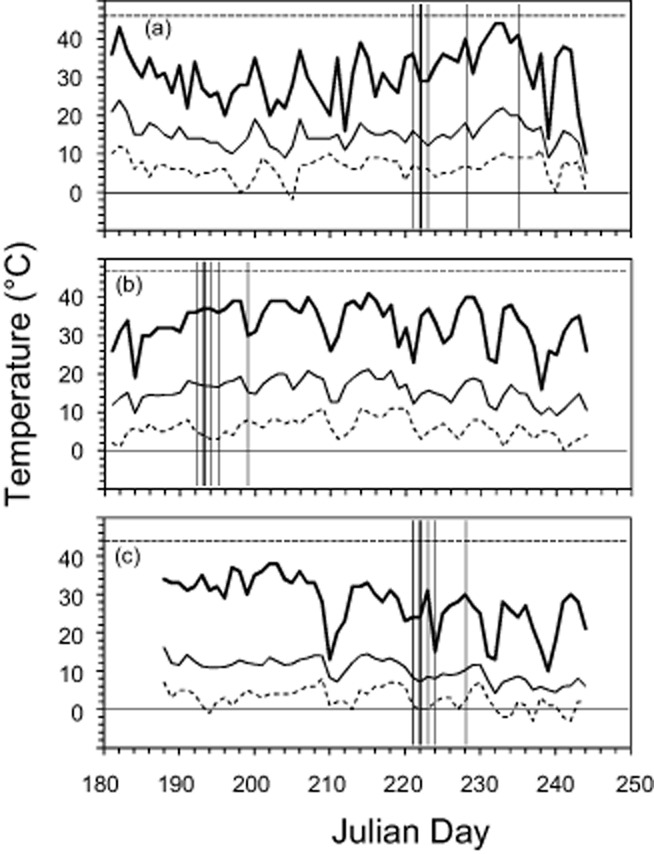
Leaf temperatures recorded in the alpine and nival plant species investigated during the summers of 2012 and 2013. (a) *Rhododendron ferrugineum* (1960 m.a.s.l.; 2012), (b) *Senecio incanus* (2165 m.a.s.l.; 2013), (c) *Ranunculus glacialis* (2560 m.a.s.l.; 2013). Data were collected in 1 min intervals using thermocouple sensors mounted onto the lower surface of leaves (*n* = 5 per species). The curves represent half-hour mean values (HHM) based on calculations of high resolution data (taken each minute). Bold lines, daily maximum HHM; regular lines, daily mean HHM; dashed lines, daily minimum HHM. The temperature thresholds of 0 °C (solid horizontal bar) and LT_5_, the temperature at which first heat-induced leaf injuries (5% of the total leaf area) were observed (dashed horizontal bar), were also shown. Vertical lines indicate the dates of the controlled heat treatment (bold) and days on which gas exchange measurements were recorded.

June 2012 was the sixth warmest June in the last 250 years (ZAMG 2013a) in Austria. At Mt. Patscherkofel (1960 m.a.s.l.; study site 1), elevated leaf temperature maxima (half-hour means; HHM) were detectable in *Rh. ferrugineum* only for short periods at the beginning, in the middle and at the end of June (e.g. 2 June 2012: 46.2 °C, 15 June 2012: 45.3 °C; Fig. 2a). Maximum leaf temperatures were much lower (36.3 °C) in July and this was followed by another episode of high temperatures, with HHMs of up to 44 °C, from August 19 to 23. The experimentally applied temperatures of 43 and 45 °C also occurred naturally in the summer of 2012. HHMs over 43 °C occurred for 5 d and over 45 °C for 2 d during the period of 1 June 2012–1 September 2012 (Table 2).

**Table 2:** Days with leaf temperature maxima >35 °C in three alpine plant species recorded in summer 2012 or 2013

*Rhododendron ferrugineum*	T_max_ [°C]	35	36	37	38	39	40	41	42	43	45	46
1960 m a.s.l; 2012; jd 153–243	f [%]	38	32	26	20	16	13	9	6	5	2	1
*Senecio incanus*	T_max_ [°C]	35	36	37	38	39	40					
2165 m.a.s.l.; 2013; jd 152–243	f [%]	39	28	17	13	5	1					
*Ranunculus glacialis*	T_max_ [°C]	35	36	37								
2560 m.a.s.l.; 2013; jd 188–243	f [%]	11	5	4								

Altitude at the study site and the investigation period (year; julian days) are given for each studied species. T_max_ are maximum half-hourly taken mean values (HHM) of temperatures in 1 °C classes. The frequency (f) describes the frequency of days at which a HHM was at least once higher than the corresponding temperature class.

#### *S. incanus* (summer 2013)

The summer of 2013 was the sixth warmest since the beginning of systematic record keeping in 1767 (ZAMG 2013b) in Austria, with long periods of high temperatures in June, July and August. The highest HHMs were recorded on 3 August 2013 (40.3 °C) and on 5 September 2013 (40.4 °C) (Fig. 2b). The experimentally applied heat stress of 44 and 46 °C did not occur naturally at the study site 2 (Mt. Patscherkofel 2165 m.a.s.l.) during the summer of 2013.

#### *Ra. glacialis* (summer 2013)

The recorded leaf temperature maxima at study site 3 (Ötztal Alps, 2560 m.a.s.l.) were not very notable, despite the high summer temperatures. Leaves of *Ra. glacialis* had a maximum HHM of 37.5 °C on 22 July 2013 (Fig. 2c). The experimentally applied heat stress of 41 and 43 °C did not occur naturally at the study site during the summer of 2013. A summary of the days with maximum HHM >35 °C for the three species and sites is provided in Table 2.

### The impact of heat on the photosynthetic parameters F_v_/F_m_, A_2000_, R_d_ and Φ

**Figure 3:**
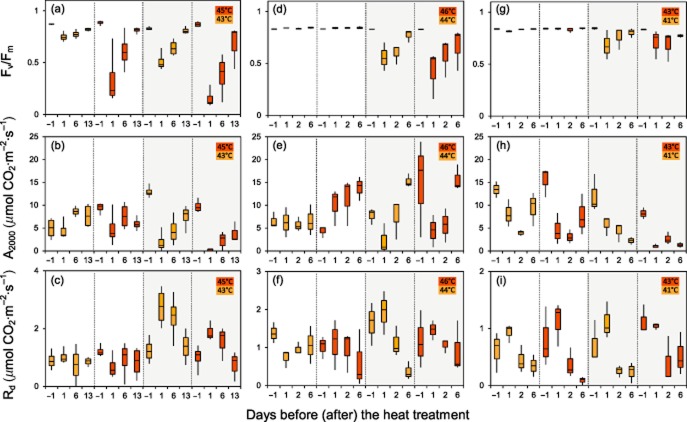
Impact of sublethal heat treatment applied *in situ* on gas exchange and various photosynthetic parameters. Parameters were derived *in situ* from photosynthetic light response curves before and after the controlled heat exposure in the heat tolerance testing system (HTTS). Different heat exposure modes are seperated by vertical dotted lines. Left column (a–c) *Rhododendron ferrugineum* (Mt. Patscherkofel, 1960 m.a.s.l., 9 August 2012), middle column (d–f) *Senecio incanus* (Mt. Patscherkofel, 2165 m.a.s.l., 11 July 2013) and right column (g–i) *Ranunculus glacialis* (Ötztal Alps, 2560 m.a.s.l., 11 August 2013). Boxplots show the time courses of the potential PSII efficiency, F_v_/F_m_ (a, d, g), of the photosynthetic CO_2_ assimilation rate at a PPFD of 2000 *μ*mol photons·m^−2^ s^−1^, A_2000_ (b, e, h), and of the dark respiration rate, R_d_ (c, f, i). The four boxes within each heat exposure mode (from left to right) represent the control value 1 d before (−1) the heat treatment followed by the values during recovery from the heat treatment. Different colours (orange, red) of boxes indicate different exposure temperatures. White background, heat treatment in the light mode; grey background, heat treatment in the dark mode.

The selected heat treatments did not cause any visible leaf damage in *S. incanus* and *Ra. glacialis* whereas a minimal level of injury occurred in *Rh. ferrugineum*. Despite the lack of visible injury, photosynthetic parameters were significantly affected by increasing temperatures in all of the investigated species. The effects of the heat treatments are summarized in Fig. 3. It should be noted that the first measurements were conducted 1 d after the applied heat stress. In addition to effects of the heat stress per se, exposure to natural environmental conditions (including solar irradiation) during the recovery period may have made an impact on the various photosynthetic parameters. Generally, when heat stress occurred under irradiation, the impact was mitigated compared with when it was applied in the dark. The percentage change in the investigated parameters in relation to reference values obtained for each leaf before the heat treatment is discussed in the succeeding text for ease of comparison between temperature and light and dark treatments. A comprehensive table of the data, including statistics, is provided as Supporting Information Appendix S1.

#### F_v_/F_m_

In the dark mode, all three species exhibited a significant reduction in F_v_/F_m_ (e.g. *Rh. ferrugineum*: 43D: 0.52 ± 0.10; 45D: 0.17 ± 0.13) during the recovery period followed by an almost complete restoration (Fig. 3a). In contrast, F_v_/F_m_ in the light mode was either completely unaffected even at the higher exposure temperature (*S. incanus*, *Ra. glacialis*) (Fig. 3d,g) or was considerably less reduced (*Rh. ferrugineum*) than in the dark mode followed by an almost complete recovery.

#### A_2000_

Heat treatment administered in the dark resulted in a reduction of A_2000_ in all three species. The effect was most pronounced in *Rh. ferrugineum* (43D: 16.8 ± 21.1%, *P* < 0.05; 45D: no positive carbon assimilation) (Fig. 3b) and *Ra. glacialis* (41D: 50.0 ± 22.8%; 43D: 13.4 ± 7.3%, *P* < 0.05); their A_2000_ value, in contrast to *S. incanus*, (Fig. 3e) did not fully recover within the investigation period (Fig. 3h). When the heat stress was administered in the light, A_2000_ transiently decreased in *Ra. glacialis* (Fig. 3h) but increased in *Rh. ferrugineum* and *S. incanus* (Fig. 3b,e). In *Ra. glacialis*, A_2000_ values recovered only in leaves exposed to heat stress in the light, whereas no recovery was observed in leaves exposed to heat stress in the dark.

#### R_d_

The heat treatment led to a transient increase in R_d_ when administered in the dark. The greatest effect was observed in *Rh. ferrugineum* (43D: 228.3 ± 36.8%; *P* < 0.05) and *Ra. glacialis* (41D: 189.51 ± 110.5%) (Fig. 3c,i). R_d_ in *S. incanus* returned to the reference level (or even lower) during the observation period (Fig. 3f), while simultaneously also exhibiting a recovery in A_2000_. These data indicate that a complete restoration of photosynthetic functions occurred. R_d_ exhibited a similar trend in *Rh. ferrugineum* and *Ra. glacialis*; however, these two species had reduced A_2000_ values at the end of the observation period. The effects of heat stress on R_d_ were less pronounced in *Rh. ferrugineum* and *S. incanus* (Fig. 3c,f) when administered in the light mode. No difference between the exposure modes could be observed in *Ra. glacialis* (Fig. 3i).

#### Φ

When the heat stress was applied in the dark mode, Φ was significantly reduced in *Rh. ferrugineum* (43D: 25.8 ± 14.7%; *P* < 0.05) and *S. incanus* (46D: 27.4 ± 23.5%; *P* < 0.05). In contrast, when the heat stress was administered in the light mode, Φ was unaffected or temporarily increased. No obvious trend was detectable in *Ra. glacialis* (Supporting Information Appendix S1).

### Diffusive conductance and PSII

**Figure 4:**
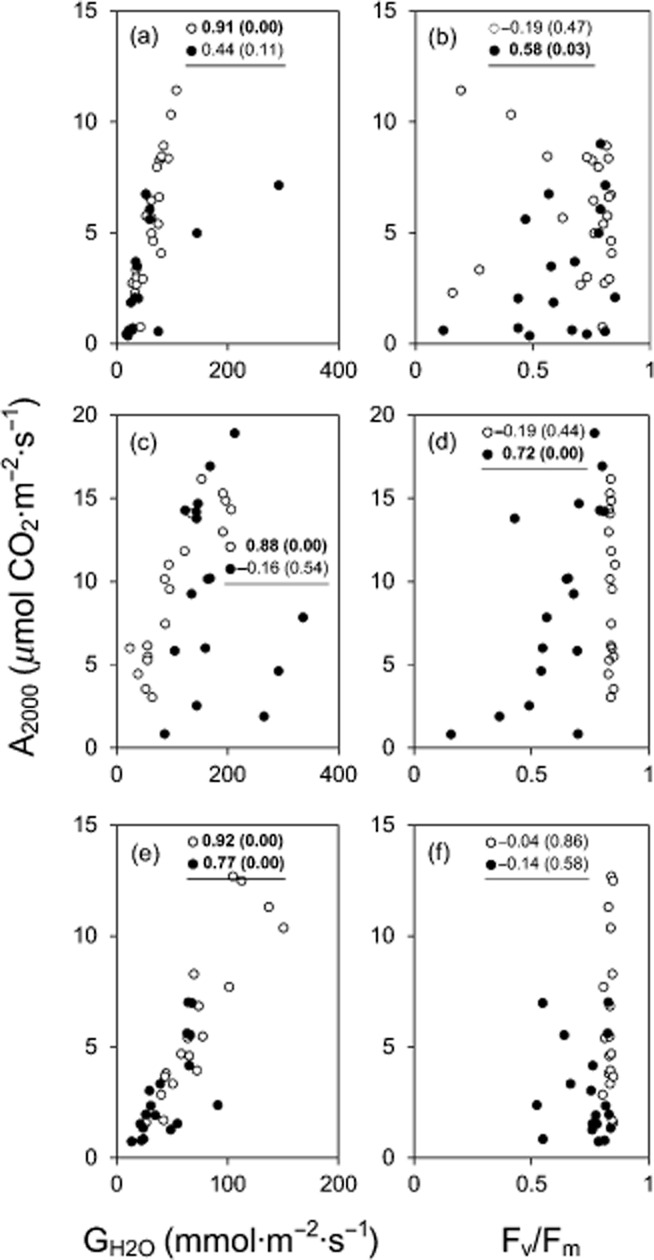
Photosynthetic performance following sublethal heat stress. Correlation diagram between the assimilation rate at 2000 *μ*mol photons·m^−2^ s^−1^, A_2000_, and (left column) the diffusive conductance G_H2O_ at PPFD 2000 *μ*mol photons·m^−2^ s^−1^ and (right column) F_v_/F_m_ for 1, 2 and 6 d after controlled heat treatment at two different sublethal temperatures in the dark mode (solid circles) or in the light mode (open circles). (a, b) *Rhododendron ferrugineum*, (c, d) *Senecio incanus*, (e, f) *Ranunculus glacialis*. Numbers: Correlation coefficient (Spearman's Rho) and significance of the correlation (values in parentheses). Significant correlations are indicated in bold letters.

During the recovery period following the heat stress, A_2000_ was reduced, particularly in leaves that were treated in the dark mode. In plants exposed to heat stress in the light mode, however, all of the investigated species showed a clear and highly significant correlation (Spearman's Rho; see Fig. 4) between A_2000_ and G_H2O_, indicating that the reduction in A_2000_ was primarily due to reduced G_H2O_ during the recovery period. No such correlation was found in plants treated in the dark mode, with the exception of *Ra. glacialis* (Fig. 4e). A_2000_ and F_v_/F_m_ were significantly correlated in plants of *Rh. ferrugineum* and *S. incanus* treated in the dark mode, indicating that the reduced A_2000_ during the recovery period was mainly caused by a reduction in F_v_/F_m_ (Fig. 4b,d). No significant correlation was detected between A_2000_ and F_v_/F_m_ (Fig. 4f) in *Ra. glacialis* and A_2000_ remained reduced even when F_v_/F_m_ was nearly completely recovered.

### T_c_ and T_c_′

**Figure 5:**
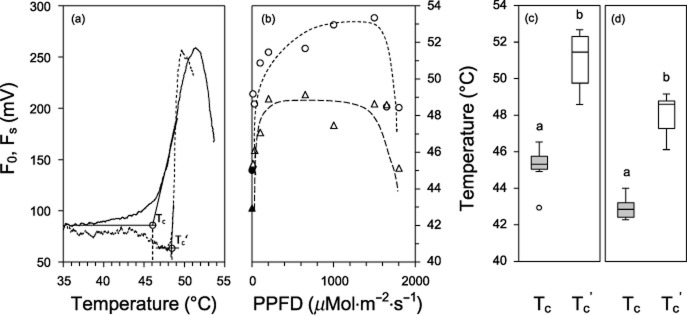
Critical high temperature threshold of PSII. (a) Typical T-F_0_ plot (temperature-basic fluorescence) for determination of T_c_ determined during controlled heating in darkness (solid line) compared with steady-state fluorescence (dotted line) recorded during controlled heating in the light (1600 *μ*mol photons·m^−2^ s^−1^). At critical temperatures (T_c_ in darkness, T_c_′ in the light), the chlorophyll fluorescence signal shows a distinct increase. (b) T_c_ and T_c_′ determined on leaves of *Senecio incanus* (circles) and *Ranunculus glacialis* (triangles) as a function of irradiation intensity. T_c_ is lowest (solid symbols, *n* = 5). T_c_′ (open symbols) increases with irradiation intensities, initially steeply in both species, then plateaus and after reaching approximately 1800 *μ*mol photons·m^−2^ s^−1^ drops again. Boxplots of T_c_ (grey boxes) and T_c_′ (white boxes; PPFD from 30 to 1650 *μ*mol photons·m^−2^ s^−1^) in leaves of (c) *S. incanus* and (d) *Ra. glacialis*. Significant differences between mean values based on the Student's *t*-test (*P* < 0.01) are indicated by different letters.

During progressive heating (1 K min^−1^) of *S. incanus* and *Ra. glacialis* plants, a rise in F_0_ was observed at a lower temperature than the rise in F_s_ (Fig. 5a). Furthermore, T_c_ was found to be 45.0 ± 1.2 °C (*n* = 5) in *S. incanus*, whereas T_c_′ was 4 K higher (49.2 °C) when the irradiation was low (10 *μ*mol photons·m^−2^ s^−1^). Above a PPFD of 100 *μ*mol photons·m^−2^ s^−1^, T_c_′ was elevated, reaching a maximum value of 53.3 °C at 1500 *μ*mol photons·m^−2^ s^−1^. The maximum difference between T_c_ and T_c_′ observed in plants of *S. incanus* was 8.3 K (Fig. 5b).

Similar results were obtained for *Ra. glacialis* with a T_c_ of 42.9 ± 0.7 °C (*n* = 5) compared with a T_c_′ of 45.4 °C at a PPFD of 10 *μ*mol photons·m^−2^ s^−1^. T_c_′ reached maximum values ranging from 47.1 to 49.2 °C, when PPFD was between 100 and 1650 *μ*mol photons·m^−2^ s^−1^. A decrease to 45.1 °C was observed, however, at a PPFD of 1800 *μ*mol photons·m^−2^ s^−1^. The maximum difference between T_c_ and T_c_′ observed was 6.3 K. In both species, T_c_ and mean T_c_′ differed significantly (*P* < 0.01) between 100 and 1650 *μ*mol photons·m^−2^ s^−1^ (Fig. 5c,d).

### Free radical scavenging activity and xanthophyll cycle pigment levels

#### Free radical scavenging activity

**Figure 6:**
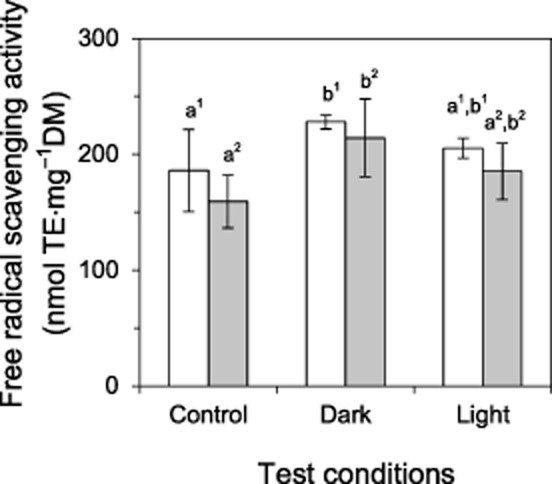
Free radical scavenging activity following controlled sublethal heat stress. Free radical scavenging activity in *Senecio incanus* (white bars) and *Ranunculus glacialis* (grey bars), expressed as trolox equivalents per mg dry mass (nmol TE·mg^−1^DM; means ± SD) immediately after the termination of an *in situ* heat stress treatment (*S. incanus*: 44 °C, *R. glacialis*: 41 °C) in the dark and light mode is shown relative to untreated plants (control). Significant differences between species (superscript numbers) are indicated by different letters (one-way anova, Duncan's test, *P* < 0.05).

Free radical scavenging activity was lowest in the non-heat-stressed control group and significantly (*P* < 0.05) increased by 34% and 46% in *S. incanus* and *Ra. glacialis*, respectively, when plants were exposed to heat stress in the dark. In contrast, when the heat treatments were administered in the light, no significant effect on free radical scavenging activity was observed (Fig. 6).

#### Xanthophyll cycle pigments

**Figure 7:**
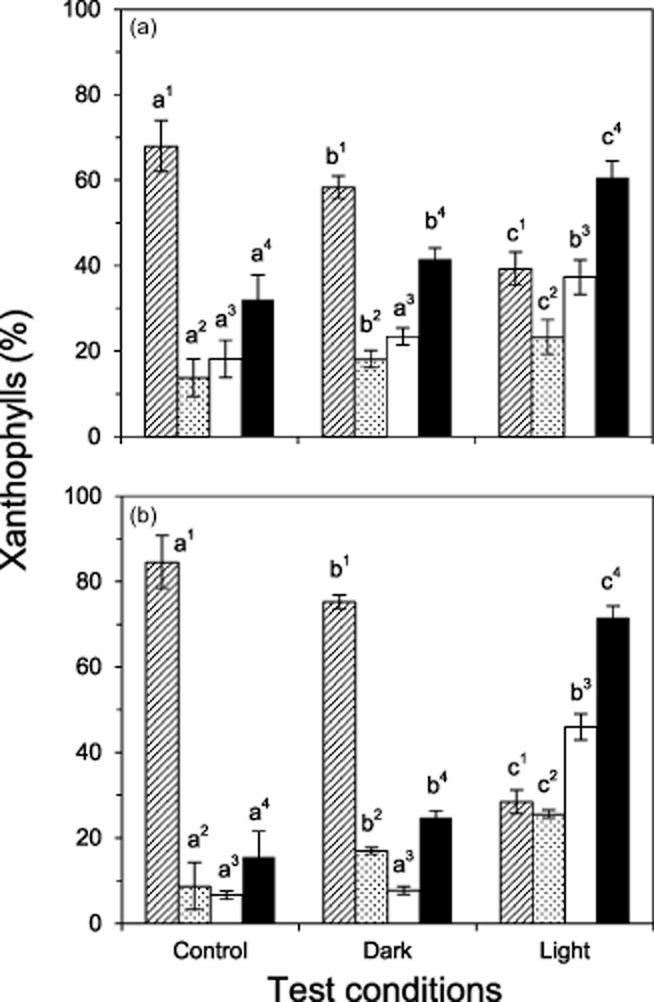
Xanthophyll cycle pigments following controlled sublethal heat stress. Violoxanthin (hatched bars), antheraxanthin (dotted bars) and zeaxanthin (white bars) in (a) *Senecio incanus* and (b) *Ranunculus glacialis*, expressed as a percentage of total xanthophyll cycle pigments, and their de-epoxidation state (A + Z)/(V + A + Z)·100% (black bars) immediately following the termination of controlled, *in situ* heat treatments (*S. incanus*: 44 °C, *Ra. glacialis*: 41 °C) in the dark mode and in the light mode are shown in comparison with untreated plants (control). Significant differences between the individual pigments (superscript numbers) are indicated by different letters (one-way anova, Duncan's test, *P* < 0.05)

The xanthophyll cycle pigments violaxanthin (V), antheraxanthin (A) and zeaxanthin (Z) were significantly (*P* < 0.05) affected by heat stress under both light and dark conditions (Fig. 7). However, the effect was most pronounced in plants exposed to heat stress in the light. The de-epoxidation status of the xanthophyll cycle pigments (V + A)/(V + A + Z) after the heat treatment was significantly (*P* < 0.05) higher in both *S. incanus* (dark mode: 41.6% ± 2.6; light mode: 60.6% ± 3.9) and *Ra. glacialis* (dark mode: 24.7% ± 1.6; light mode: 71.5% ± 2.8) relative to the non-stressed control plants (*S. incanus*: 32.0% ± 5.9; *Ra. glacialis*: 15.4% ± 6.2). Other photosynthetic pigments (chlorophyll *a* and *b*, *β*-carotene, neoxanthin and lutein) did not change significantly in response to the heat treatment, with the exception of *β*-carotene and lutein, which significantly (*P* < 0.05) increased by 15% and 28%, respectively, in *S. incanus* after heat treatment in the dark compared with heat treatment in the light. These data are in agreement with the finding that free radical scavenging activity increased for *S. incanus* in response to the heat treatment when it was administered in the dark.

## Discussion

### Residual effects of sublethal heat stress on photosynthesis

The effect of global warming on the climate of the European Alps is rather evident, however, few studies have been conducted on the effects of heat stress on alpine plants (e.g. Larcher & Wagner 1976, Gauslaa 1984, Neuner *et al*. 1999, Buchner & Neuner 2001, 2003; Marcante *et al*. 2014). It is important to note that, in these few studies, heat treatments were typically applied in the dark to detached plant material in a laboratory setting. Such experimental conditions do not provide information on: (1) the effect of solar irradiation on the response of plants to heat stress, and; (2) the ability of plants in their natural habitat to recover from the deleterious impacts of the heat stress. In the current study, the HTTS was used, which allowed for the *in situ* evaluation of the impacts of sublethal heat stress on photosynthetic performance, photosynthetic pigments and free radical scavenging activity in three high mountain species. Results indicated that the alpine plant species studied tolerated heat stress much better in the light, at levels that corresponded to natural solar irradiation. By contrast, after the termination of the heat stress, the residual effects on F_v_/F_m_, A_2000_, Φ and R_d_ were greater in plants that had been exposed to the heat stress in the dark. In this context, it again must be pointed out that the presented residual effects may not only be due to the heat stress per se but may show also impacts made by exposure to natural environmental conditions during recovery. Further, it was demonstrated that the heat response of the investigated plant species also has species-specific components.

The heat stress-induced decrease in A_2000_ in the light in all three species can be primarily attributed to a reduction in G_H2O_, which lasted for a long time (Fig. 4). Abiotic stress factors, such as salinity or drought, are known to alter photosynthetic gas exchange via their effect on stomatal and non-stomatal parameters (see Ashraf & Harris 2013). The strong correlation between A_2000_ and G_H2O_ after exposure to heat stress in the light mode indicates that the reduction in A_2000_ during the recovery period was mainly due to stomatal parameters.

In contrast, the effect of heat stress in the dark on A_2000_ was primarily due to the reduced efficiency of PSII. It is evident that stomatal conductance, photosynthesis and transpiration interact in a complex manner (Farquhar & Sharkey 1982; Tuzet *et al*. 2003) and that stomatal conductance may be correlated with actual photosynthetic capacity (Wong *et al*. 1979). However, the long-term effect of heat stress, when applied *in situ* in the light, on G_H2O_ with a concomitant decrease in photosynthetic performance, does not appear to have been previously reported in the literature. Our results demonstrated that within either 6 or 13 d, depending on species, F_v_/F_m_ recovered slowly in all three species, when the heat stress was administered in the dark, whereas A_2000_ recovered fully in *S. incanus*, slowly in *Rh. ferrugineum* and hardly at all in *Ra. glacialis*. Hence, the HTTS allowed us to gain deeper insights into the remarkable and species-specific ability of high mountain plants to recover photosynthetic performance following a severe heat stress.

The data in Figs 4 and 5 offer a possible explanation for the contribution of PSII to the protective effect of light in heat-stressed plants. T_c_^′^, the critical high temperature threshold for PSII, increased to higher temperatures during heat exposure with increasing irradiation. The increase in T_c_^′^ supports the observation that PSII was more stable when heat stress occurred in the light, resulting in smaller residual effects on photosynthesis after the heat stress was terminated. Nevertheless, the heat treatments *in situ* were done at lower temperatures than the critical temperature thresholds, potential aftereffects of critically high temperatures remain to be examined. As observed in *Rh. ferrugineum*, even low irradiation during heat stress (e.g. mean PPFD 261 *μ*mol photons·m^−2^ s^−1^) was sufficient to elicit a protective effect. These findings also corroborate earlier results obtained in laboratory experiments which indicated the potential protective role of irradiation, even at very low intensities, on the stability of PSII during heat stress (Weis 1982). The mechanisms responsible for the increased heat stability of PSII under irradiation are complex and still not fully understood. The stability of PSII function at high temperatures may not only be affected by light but also by osmotic potential and sugar concentration (Hüve *et al*. 2006), as well as by any process leading to an impairment of the electron transport chain.

Importantly, the observed protective effect of irradiation during heat stress on photosynthesis need not only be due to the increased stability of PSII but could also be explained by other mechanisms. It is conceivable that the heat treatment may have reduced the activation status of rubisco because of the heat sensitivity of rubisco activase (Salvucci *et al*. 2001), which is known to play a key role in limiting photosynthesis during heat stress (Salvucci & Crafts-Brandner 2004) and during recovery from exposure to high temperature (Kim & Portis 2005). As the activation state of rubisco activase is also affected by light-mediated changes of the stromal ATP/ADP ratio (Portis *et al*. 2008), it can be assumed that heat stress applied in the dark mode could result in a stronger decrease in rubisco activase activity compared with heat stress applied in the light mode. The exposure of the plants from darkness to high irradiation intensities after the termination of the heat treatment could have induced photoinhibition and photodamage because of at least a temporary partial inactivation of the Calvin cycle. In turn, this partial inactivation may have resulted in a reduction in F_v_/F_m_ and A_2000_. In this scenario, the observed reduction in F_v_/F_m_ could be interpreted as a consequence resulting from the impairment of the Calvin cycle, rather than because of the limited heat stability of PSII as a primary cause. The relevance of rubisco activase and non-photochemical fluorescence quenching (NPQ) in relation to rapidly increasing irradiation was recently demonstrated by Yamori *et al*. (2012). When rice plants were suddenly subjected to high irradiation, the electron transport rate (ETR) increased much faster in genetically modified plants overexpressing rubisco activase compared with wild-type plants, while NPQ was lower. Unfortunately, our field measurements did not provide the data necessary to perform a similar analysis.

During the first hour after the heat treatment was terminated, leaves of *Rh. ferrugineum* were exposed to moderate irradiation (PPFD approximately 500 *μ*mol photons·m^−2^ s^−1^ with maxima approximately 1000 *μ*mol photons·m^−2^ s^−1^), while *S. incanus* was continuously exposed to approximately 1000 *μ*mol photons·m^−2^ s^−1^ and *Ra. glacialis* was exposed to approximately 2000 *μ*mol photons·m^−2^ s^−1^. This may also have potentially affected the results obtained in the present study. High mountain plants have the potential to cope with the rapidly changing environmental conditions, including irradiation and temperature. PPFD can rapidly fluctuate between very low levels (<50 *μ*mol photons·m^−2^ s^−1^) when the sky is clouded, and >2500 *μ*mol photons·m^−2^ s^−1^ in full sunlight. Photosynthetic carbon assimilation in some species is often not saturated at a PPFD of 2000 *μ*mol photons·m^−2^ s^−1^ (Streb & Cornic 2012). Therefore, such effects of the sudden exposure to high levels of irradiation immediately after the heat treatment seem rather unlikely but cannot be ruled out entirely, and may have to some minor degree influenced the results. Also, the accumulation of heat shock proteins (HSP) which may be induced in response to a combination of high temperature and light, as demonstrated in *Solidago altissima* (Barua & Heckathorn 2006), could have contributed to the minor residual effects of heat stress in the light mode. Regardless of the mechanism, irradiation alters the effect of heat stress on PSII. When the heat stress is applied in the light, PSII appears to play a pivotal role in the response of photosynthesis to heat stress. This is due to (1) photoinhibition, which is increased at higher temperatures (see Streb *et al*. 2003, Dongsansuk *et al*. 2005) and (2) a shift in T_c_^′^ towards higher temperatures.

### Protective effect of natural solar irradiation

The protective effect of irradiation in plants subjected to heat stress has fascinated plant scientists for a long time, and yet the mechanistic basis of the protection still remains to be elucidated. As previously stated, the protective role of irradiation is not likely the result of a single mechanism but rather results from a combination of several factors, including the activation state of rubisco, ROS-scavenging activity and HSP accumulation. ROS levels often increase in response to heat stress and ROS-scavenging mechanisms are important in protecting plants against high temperature stress (Allakhverdiev *et al*. 2008). In addition, ROS are also intricately involved in oxidative signalling (Foyer & Noctor 2009). In the present study, free radical scavenging activity was used as an overall indicator of the level of stress. The increase of free radical scavenging activity was significant when heat was applied in the dark, confirming that the heat treatment caused more stress in the dark than in the light. It is also conceivable that an earlier onset of ROS signalling may have taken place in dark-treated leaves than in the light-mode leaves, and led to a more rapid stress response, resulting in a more extensive up-regulation or activation of ROS-scavenging molecules. The stable free radical DPPH reacts with many antioxidants, albeit with different kinetics (Mishra *et al*. 2012), and so the DPPH assay does not allow one to draw conclusions about the composition of the ROS-scavenging components. It will be interesting to characterize the role of specific ROS-scavenging compounds in the response to heat stress applied in darkness versus in lightness (especially at different levels of irradiation), but this was not an aim of the present study.

Havaux *et al*. (1999) reported a potential connection between the photoprotection of PSII and irradiation-induced inter-thylakoid acidification, resulting in the stabilization of the thylakoid membrane and PSII reaction centres. Additionally, acidification of the thylakoid lumen also activates violaxanthin de-epoxidase, thus activating the xanthophyll cycle. The formation of ROS can be partly avoided by non-photochemical quenching (NPQ) of excess light and the xanthophyll cycle is an important part of NPQ. In accordance with earlier results (Yin *et al*. 2010; Dongsansuk *et al*. 2005), heat treatment applied in the light led to a significant increase of xanthophyll cycle activity in both species investigated. Streb *et al*. (2003) also reported increased xanthophyll cycle activity in *Ra. glacialis* leaves at 38 °C under strong irradiation. Increased xanthophyll cycle activity in the heat-exposed plants likely contributed to the protection of the photosynthetic apparatus from heat damage and may at least partly explain the better survival of leaves exposed to heat stress in the light. Nevertheless, the xanthophyll cycle was also activated when heat stress was applied in the dark, although to a much lesser extent. Investigating the mechanism underlying the activation of the xanthophyll cycle in the dark was not a key objective in the present study. However, it is interesting to note that Fernández-Marín *et al*. (2010, 2011) also found that the enzyme violaxanthin de-epoxidase was activated in the lichen, *Lobularia pulmonaria*, and in the brown seaweed, *Pelvetia canaliculata*, in the dark in response to desiccation or high temperature. Furthermore, violaxanthin de-epoxidation was also induced in response to heat stress at very low irradiation (12 *μ*mol photons m^−1^·s^−1^) in wheat seedlings (Ilik *et al*. 2010). In contrast, Abramchik *et al*. (2013) reported that a 3 h heat treatment at 44 °C and a PPFD of 64 *μ*mol photons·m^−2^ s^−1^ did not affect xanthophyll cycle activity in seedlings of different *Triticale* cultivars. Zeaxanthin may also protect thylakoid membranes from lipid peroxidation not only by quenching of ^1^Chl but also by NPQ-independent mechanisms (see Havaux & Niyogi 1999; Johnson *et al*. 2007; Jahns & Holzwarth 2012). In summary, heat stress applied in the light, and to a lesser extent also in the dark, led to violaxanthin de-epoxidation, resulting in the formation of antheraxanthin and zeaxanthin, which may have contributed to the protection of the photosynthetic apparatus.

Finally, Yamauchi *et al*. (2011) showed that in the light, heat stress can cause preferential excitation of PSI and enhance thermal dissipation and light-driven cyclic electron flow around PSI. This can protect PSII from damage because of a backflow of reducing power from the stroma to PSII. In turn, this backflow may cause an overreduction of plastoquinone and result in damage to the D1 protein of PSII reaction centres (Marutani *et al*. 2012).

It is generally accepted that two or more stress factors are more stressful to an organism than one factor alone (Kranner *et al*. 2010). On their own, light and heat both have the potential to cause photoinhibition and photodestruction. Therefore, one could intuitively assume that the combination of high light and heat stress will cause more damage than heat stress in the dark. However, this assumption is fraught with difficulties because for a plant, the absence of light is stressful. Few authors have considered the potential protective effects of irradiation on photosynthetic functions, including the reduced inactivation of CO_2_-fixation in *Spinacia oleracea* chloroplasts (Weis 1982) and the enhanced PSII function in barley leaves (Havaux & Tardy 1997; Kalituho *et al*. 2003), when plants were subjected to heat stress. Havaux *et al*. (1999) suggested that light may protect photochemical activity from inactivation by heat. Our results largely support these observations, but they also demonstrate that recovery of heat-treated leaves under field conditions is highly dynamic and species-specific.

### Relevance of the study to current and future microclimate conditions of alpine plants

In the European Alps, particularly at high elevation, the increase in atmospheric temperature was found to be doubled than what was estimated for global warming in general (1890–1998, +1.1 °C versus +0.55 °C; Böhm *et al*. 2001). If this trend continues, heat stress will become an increasingly significant factor for alpine plants.

The present study illustrates that under current climatic conditions, photosynthesis of individual alpine plant species can already be temporarily reduced by naturally occurring heat waves. Global temperatures are predicted to increase erratically (IPCC 2013), with more frequent heat waves in Europe (Schär *et al*. 2004), which will affect photosynthetic performance of high mountain plants accordingly. However, this will not necessarily lead to a widespread extinction of alpine plants, because due to the dense thermal microhabitat mosaics that are characteristic of high elevation sites, most plant species can readily ‘escape’ the detrimental thermal regime (Scherrer & Körner 2011). Furthermore, global climate change will undoubtedly promote an upward shift of certain species to higher elevations (Grabherr *et al*. 1994; Holzinger *et al*. 2008; Pauli *et al*. 2012). We demonstrated that the temperatures applied to alpine plants in the current study are realistic and may occur under present climate conditions. Alpine dwarf-shrub heaths can exhibit canopy temperatures that are significantly higher than air temperature (Larcher & Wagner 2010), and plants in the sub-alpine zone may be at a higher risk of being injured by heat stress than nival species. Based on the available data, it seems likely that *Ra. glacialis* but not *S. incanus* will be negatively affected if leaf temperatures rise to between 41 and 46 °C (as in our experiments). It is expected that mean air temperature will increase by 0.3–0.4 K per decade in the European Alps from the present through 2100 (Gobiet *et al*. 2014). Therefore, it can be assumed that any further increases in temperature, or more frequent heat waves, will have the potential to reduce photosynthetic performance for considerable periods of time in a very species-specific manner. In the long term, such a changing thermal environment will further increase the pressure on heat-sensitive and less-competitive species and favour species migration, which can already be observed in the European Alps today.

## SUPPORTING INFORMATION

Appendix S1 Effects of controlled heat treatment administered *in situ* on photosynthetic parameters in selected alpine plant species during the recovery period following the termination of the heat treatment. Values are expressed as mean percentage increase/decrease compared with reference values (100%) as determined before the heat treatment. A_2000_ assimilation rate at PPFD 2000 *μ*mol photons·m^−2^ s^−1^, R_d_ dark respiration rate, Φ maximum quantum efficiency determined as the positive linear slope in the initial phase of the light response curve, F_v_/F_m_ potential efficiency of PSII. Exposure mode: numbers: exposure temperature [°C], L light-mode, D dark-mode. For each day after the heat treatment, significant differences between treatments (anova, Duncan test, *P* < 0.05) are indicated by different characters. Significant differences compared with the reference value as determined before the heat treatment are indicated by asterisks (*t*-test, *P* < 0.05).

## References

[b1] Abramchik LM, Kabashnikova LF, Savchenko GE (2013). The xanthophyll pigments and abscisic acid under heat stress in green seedlings of short- and long-stem cultivars of triticale. Genetics and Plant Physiology.

[b2] Allakhverdiev SI, Kreslavski VD, Klimov VV, Los DA, Carpentier R, Mohanty P (2008). Heat stress: an overview of molecular responses in photosynthesis. Photosynthesis Research.

[b3] Ashraf M, Harris PJC (2013). Photosynthesis under stressful environments. An overview. Photosynthetica.

[b4] Baker NR (2008). Chlorophyll fluorescence: a probe of photosynthesis in vivo. Annual Review of Plant Biology.

[b5] Barua D, Heckathorn SA (2006). The interactive effects of light and temperature on heat-shock protein accumulation in *Solidago altissima* (Asteraceae) in the field and laboratory. American Journal of Botany.

[b6] Bauer H, Larcher W, Cooper JP, Walker RB (1975). Influence of temperature stress on CO_2_-gas exchange. Photosynthesis and Productivity in Different Environment.

[b7] Bilger HW, Schreiber U, Lange OL (1984). Determination of leaf heat resistance: comparative investigation of chlorophyll fluorescence changes and tissue necrosis methods. Oecologia.

[b8] Bilger W, Björkman O (1990). Role of the xanthophyll cycle in photoprotection elucidated by measurements of light-induced absorbance changes, fluorescence and photosynthesis in leaves of *Hedera canariensis*. Photosynthesis Research.

[b9] Böhm R, Auer I, Brunetti M, Maugeri M, Nanni T, Schöner W (2001). Regional temperature variability in the European Alps: 1760–1998 from homogenized instrumental time series. International Journal of Climatology.

[b10] Brand-Williams W, Cuvelier ME, Berset C (1995). Use of a free radical method to evaluate antioxidant activity. LWT – Food Science and Technology.

[b11] Braun V, Buchner O, Neuner G (2002). Thermotolerance of photosystem 2 of three alpine plant species under field conditions. Photosynthetica.

[b12] Buchner O, Neuner G (2001). Determination of heat tolerance: a new equipment for field measurements. Journal of Applied Botany – Angewandte Botanik.

[b13] Buchner O, Neuner G (2003). Variability of heat tolerance in alpine plant species measured at different altitudes. Arctic, Antarctic and Alpine Research.

[b14] Buchner O, Karadar M, Bauer I, Neuner G (2013). A novel system for *in situ* determination of heat tolerance of plants: first results on alpine dwarf shrubs. Plant Methods.

[b15] Carpentier R (1999). Effect of high-temperature stress on the photosynthetic apparatus. Handbook of Plant and Crop Stress.

[b16] Demmig-Adams B, Allen JF, Govindjee, Beatty JT, Gest H Linking the xanthophyll cycle with thermal energy dissipation. Discoveries in Photosynthesis.

[b17] Dongsansuk A, Lütz C, Neuner G (2005). Effects of temperature and irradiance on quantum yield of PSII photochemistry and xanthophyll cycle in a tropical and a temperate species. Photosynthetica.

[b18] Ducruet JM, Peeva V, Havaux M (2007). Chlorophyll thermofluorescence and thermoluminescence as complementary tools for the study of temperature stress in plants. Photosynthesis Research.

[b19] Farquhar GD, Sharkey TD (1982). Stomatal conductance and photosynthesis. Annual Review of Plant Physiology.

[b20] Fernández-Marín B, Becerril JM, García-Plazaola JI (2010). Unravelling the roles of desiccation-induced xanthophyll cycle activity in darkness: a case study in *Lobaria pulmonaria*. Planta.

[b21] Fernández-Marín B, Míguez F, Becerril JM, García-Plazaola JI (2011). Activation of violaxanthin cycle in darkness is a common response to different abiotic stresses: a case study in *Pelvetia canaliculata*. BMC Plant Biology.

[b22] Foyer CH, Noctor G (2009). Redox regulation in photosynthetic organisms: signaling, acclimation, and practical implications. Antioxidants & Redox Signaling.

[b23] Fukumoto LR, Mazza G (2000). Assessing antioxidant and prooxidant activities of phenolic compounds. Journal of Agricultural and Food Chemistry.

[b24] Gauslaa Y (1984). Heat resistance and energy budget in different Scandinavian plants. Holarctic Ecology.

[b25] Gobiet A, Kotlarski S, Beniston M, Heinrich G, Rajczak J, Stoffel M (2014). 21st century climate change in the European Alps – a review. Science of the Total Environment.

[b26] Grabherr G, Gottfried M, Pauli H (1994). Climate effects on mountain plants. Nature.

[b27] Havaux M, Niyogi KK (1999). The violaxanthin cycle protects plants from photooxidative damage by more than one mechanism. Proceedings of the National Academy of Sciences of the United States of America.

[b28] Havaux M, Tardy F (1997). Thermostability and photostability of photosystem II in leaves of the Chlorina-f2 barley mutant deficient in light-harvesting chlorophyll a/b protein complexes. Plant Physiology.

[b29] Havaux M, Greppin H, Strasser RJ (1991). Functioning of photosystems I and II in pea leaves exposed to heat stress in the presence or absence of light. Planta.

[b30] Havaux M, Greppin H, Strasser RJ (1999). Functioning of photosystem I and II in pea leaves exposed to heat stress in the presence or absence of light; analysis using *in-vivo* fluorescence, absorbance, oxygen and photoacoustic measurements. Planta.

[b31] Holzinger B, Hülber K, Camenisch M, Grabherr G (2008). Changes in plant species richness over the last century in the eastern Swiss Alps: elevational gradient, bedrock effects and migration rates. Plant Ecology.

[b32] Hüve K, Bichele I, Tobias M, Niinemets U (2006). Heat sensitivity of photosynthetic electron transport varies during the day due to changes in sugars and osmotic potential. Plant, Cell & Environment.

[b33] Ilik P, Kotabova E, Spundova M, Novak O, Kana R, Strzałka K (2010). Low-light-induced violaxanthin de-epoxidation in shortly preheated leaves: uncoupling from delta pH-dependent nonphotochemical quenching. Photochemistry and Photobiology.

[b34] Stocker TF, Qin D, Plattner GK, Tignor M, Allen SK, Boschung J, Nauels A, Xia Y, Bex V, Midgley PM, IPCC (2013).

[b35] Jahns P, Holzwarth AR (2012). The role of the xanthophyll cycle and of lutein in photoprotection of photosystem II. Biochimica et Biophysica Acta (BBA) – Bioenergetics.

[b36] Johnson MP, Havaux M, Triantaphylides C, Ksas B, Pascal AA, Robert B, Horton P (2007). Elevated zeaxanthin bound to oligomeric LHCII enhances the resistance of *Arabidopsis* to photooxidative stress by a lipid-protective, antioxidant mechanism. Journal of Biological Chemistry.

[b37] Kalituho LN, Pshybytko NL, Kabashnikova LF, Jahns P (2003). Photosynthetic apparatus and high temperature: role of light. Bulgarian Journal of Plant Physiology.

[b38] Kim K, Portis AR (2005). Temperature dependence of photosynthesis in *Arabidopsis* plants with modifications in rubisco activase and membrane fluidity. Plant and Cell Physiology.

[b39] Körner C (2003). Alpine Plant Life. Functional Plant Ecology of High Mountain Ecosystems.

[b40] Körner C, Cochrane P (1983). Influence of plant physiognomy on leaf temperature on clear midsummer days in the Snowy Mountains, south-eastern Australia. Acta Oecologia/Oecologia Plantarum.

[b41] Körner C, Larcher W (1988). Plant life in cold climates. Symposia of the Society for Experimental Biology.

[b42] Kranner I, Minibayeva FV, Beckett RP, Seal CE (2010). What is stress? Concepts, definitions and applications in seed science: Tansley review. New Phytologist.

[b43] Krause GH, Winter K, Krause B, Jahns P, García M, Aranda J, Virgo A (2010). High-temperature tolerance of a tropical tree, *Ficus insipida*: methodological reassessment and climate change considerations. Functional Plant Biology.

[b44] Kreeb KH, Kreeb KH (1990). Hitzeresistenz. Methoden zur Pflanzenökologie und Bioindikation.

[b45] Larcher W, Wagner J (1976). Temperaturgrenzen der CO_2_ Aufnahme und Temperaturresistenz der Blätter von Gebirgspflanzen im vegetationsaktiven Zustand. Oecologia Plantarum.

[b46] Larcher W, Wagner J (2010). Temperatures in the life zones of the Tyrolean Alps. Sitzungsbericht der Österreichischen Akademie der Wissenschaften, mathematisch-naturwissenschaftliche Klasse.

[b47] Larcher W, Heber U, Larcher W, Santarius KA, Precht J, Christophersen H, Hensel H (1973). Limiting temperatures for life functions. Temperature and Life.

[b48] Larcher W, Wagner J, Lütz C (1997). The effect of heat on photosynthesis, dark respiration and cellular ultrastructure of the arctic-alpine psychrophyte *Ranunculus glacialis*. Photosynthetica.

[b49] Latowski D, Kuczyńska P, Strzałka K (2011). Xanthophyll cycle – a mechanism protecting plants against oxidative stress. Redox Report.

[b50] Laureau C, Bligny R, Streb P (2011). The significance of glutathione for photoprotection at contrasting temperatures in the alpine plant species *Soldanella alpina* and *Ranunculus glacialis*. Physiologia Plantarum.

[b51] Marcante S, Erschbamer B, Buchner O, Neuner G (2014). Heat tolerance of early developmental stages of glacier foreland species in the growth chamber and in the field. Plant Ecology.

[b52] Marutani Y, Yamauchi Y, Kimura Y, Mizutani M, Sugimoto Y (2012). Damage to photosystem II due to heat stress without light-driven electron flow: involvement of enhanced introduction of reducing power into thylakoid membranes. Planta.

[b53] Mishra K, Ojha H, Chaudhury NK (2012). Estimation of antiradical properties of antioxidants using DPPH assay: a critical review and results. Food Chemistry.

[b54] Neuner G, Lütz C, Buchner O (2012). Dynamics of tissue heat tolerance and thermotolerance of PS II in alpine plants. Plants in Alpine Regions. Cell Physiology of Adaption and Survival Strategies.

[b55] Neuner G, Pramsohler M (2006). Freezing and high temperature thresholds of photosystem 2 compared to ice nucleation, frost and heat damage in evergreen subalpine plants. Physiologia Plantarum.

[b56] Neuner G, Buchner O, Braun V, Taschler D (1999). Leaf rosette closure in the alpine rock species *Saxifraga paniculata* Mill.: significance for survival of drought and heat under high irradiation. Plant, Cell & Environment.

[b57] Neuner G, Buchner O, Braun V (2000). Short-term changes in heat tolerance in the alpine cushion plant *Silene acaulis* ssp. excapa [All.] J. Braun at different altitudes. Plant Biology.

[b58] Pauli H, Gottfried M, Dullinger S, Abdaladze O, Akhalkatsi M, Alonso JLB, Grabherr G (2012). Recent plant diversity changes on Europe's mountain summits. Science.

[b59] Pfeifhofer HW, Willfurth R, Zorn M, Varma A, Kranner I, Kranner I, Beckett RP (2002). Analysis of chlorophylls, carotenoids, and tocopherols in Lichens. Protocols in Lichenology Culturing, Biochemistry, Ecophysiology and Use in Biomonitoring.

[b60] Portis AR, Li C, Wang D, Salvucci ME (2008). Regulation of rubisco activase and its interaction with rubisco. Journal of Experimental Botany.

[b61] Salisbury FB, Spomer GG (1964). Leaf temperatures of alpine plants in the field. Planta.

[b62] Salvucci ME, Crafts-Brandner SJ (2004). Inhibition of photosynthesis by heat stress: the activation state of rubisco as a limiting factor in photosynthesis. Physiologia Plantarum.

[b63] Salvucci ME, Osteryoung KW, Crafts-Brandner SJ, Vierling E (2001). Exceptional sensitivity of rubisco activase to thermal denaturation *in vitro* and *in vivo*. Plant Physiology.

[b64] Schär C, Vidale PL, Lüthi D, Frei C, Häberli C, Liniger MA, Appenzeller C (2004). The role of increasing temperature variability in European summer heatwaves. Nature.

[b65] Scherrer D, Körner C (2011). Topographically controlled thermal-habitat differentiation buffers alpine plant diversity against climate warming. Journal of Biogeography.

[b66] Schreiber U, Berry JA (1977). Heat-induced changes of chlorophyll fluorescence in intact leaves correlated with damage of the photosynthetic apparatus. Planta.

[b67] Streb P, Lütz C, Cornic G (2012). Photosynthesis and antioxidative protection in alpine herbs. Plants in Alpine Regions. Cell Physiology of Adaption and Survival Strategies.

[b68] Streb P, Feierabend J, Bligny R (1997). Resistance to photoinhibition of photosystem II and catalase and antioxidative protection in high mountain plants. Plant, Cell & Environment.

[b69] Streb P, Aubert S, Bligny R (2003). High temperature effects on light sensitivity in the two high mountain plant species *Soldanella alpina* (L.) and *Ranunculus glacialis* (L.). Plant Biology.

[b70] Streb P, Josse E, Gallouet E, Baptist F, Kuntz M, Cornic G (2005). Evidence for alternative electron sinks to photosynthetic carbon assimilation in the high mountain plant species *Ranunculus glacialis*. Plant, Cell & Environment.

[b71] Tuzet A, Perrier A, Leuning R (2003). A coupled model of stomatal conductance, photosynthesis and transpiration. Plant, Cell & Environment.

[b72] Weis E (1982). Influence of light on the heat sensitivity of the photosynthetic apparatus in isolated spinach chloroplasts. Plant Physiology.

[b73] Weis E, Berry JA (1988). Plants and high temperature stress. Symposia of the Society for Experimental Biology.

[b74] Weng JH, Lai MF (2005). Estimating heat tolerance among plant species by two chlorophyll fluorescence parameters. Photosynthetica.

[b75] Wildi B, Lütz C (1996). Antioxidant composition of selected high alpine plant species from different altitudes. Plant, Cell & Environment.

[b76] Willits DH, Peet MM (2001). Measurement of chlorophyll fluorescence as a heat stress indicator in tomato: laboratory and greenhouse comparisons. Journal of the American Society for Horticultural Science.

[b77] Wong SC, Cowan IR, Farquahr GD (1979). Stomatal conductance correlates with photosynthetic capacity. Nature.

[b78] Yamauchi Y, Kimura Y, Akimoto S, Marutani Y, Mizutani M, Sugimoto Y (2011). http://hdl.handle.net/10101/npre.2011.6168.1.

[b79] Yamori W, Masumoto C, Fukayama H, Makino A (2012). Rubisco activase is a key regulator of non-steady-state photosynthesis at any leaf temperature and, to a lesser extent, of steady-state photosynthesis at high temperature: regulation of photosynthesis by rubisco activase. The Plant Journal.

[b80] Yin Y, Li S, Liao W, Lu Q, Wen X, Lu C (2010). Photosystem II photochemistry, photoinhibition, and the xanthophyll cycle in heat-stressed rice leaves. Journal of Plant Physiology.

[b81] ZAMG (2013a). http://www.zamg.ac.at/cms/de/klima/news/sechstwaermster-juni-seit-messbeginn.

[b82] ZAMG (2013b). http://www.zamg.ac.at/cms/de/klima/news/sommer-2013.

