# Supplementary material for: Application of heat stress in situ demonstrates a protective role of irradiation on photosynthetic performance in alpine plants
Source: Plant Cell Environ. 2014 Nov 28;38(4):812–26. doi: 10.1111/pce.12455 (PMC4407927; doi:10.1111/pce.12455)
Supplement: Appendix S1 — Effects of controlled heat treatment administered in situ on photosynthetic parameters in selected alpine plant species during the recovery period following the termination of the heat treatment. Values are expressed as mean percentage increase/decrease compared with reference values (100%) as determined before the heat treatment. A2000 assimilation rate at PPFD 2000 μmol photons·m−2 s−1, Rd dark respiration rate, Φ maximum quantum efficiency determined as the positive linear slope in the initial phase of the light response curve, Fv/Fm potential efficiency of PSII. Exposure mode: numbers: exposure temperature [°C], L light-mode, D dark-mode. For each day after the heat treatment, significant differences between treatments (anova, Duncan test, P < 0.05) are indicated by different characters. Significant differences compared with the reference value as determined before the heat treatment are indicated by asterisks (t-test, P < 0.05). [file pce0038-0812-sd1.zip › pce12445_sf1.pdf]

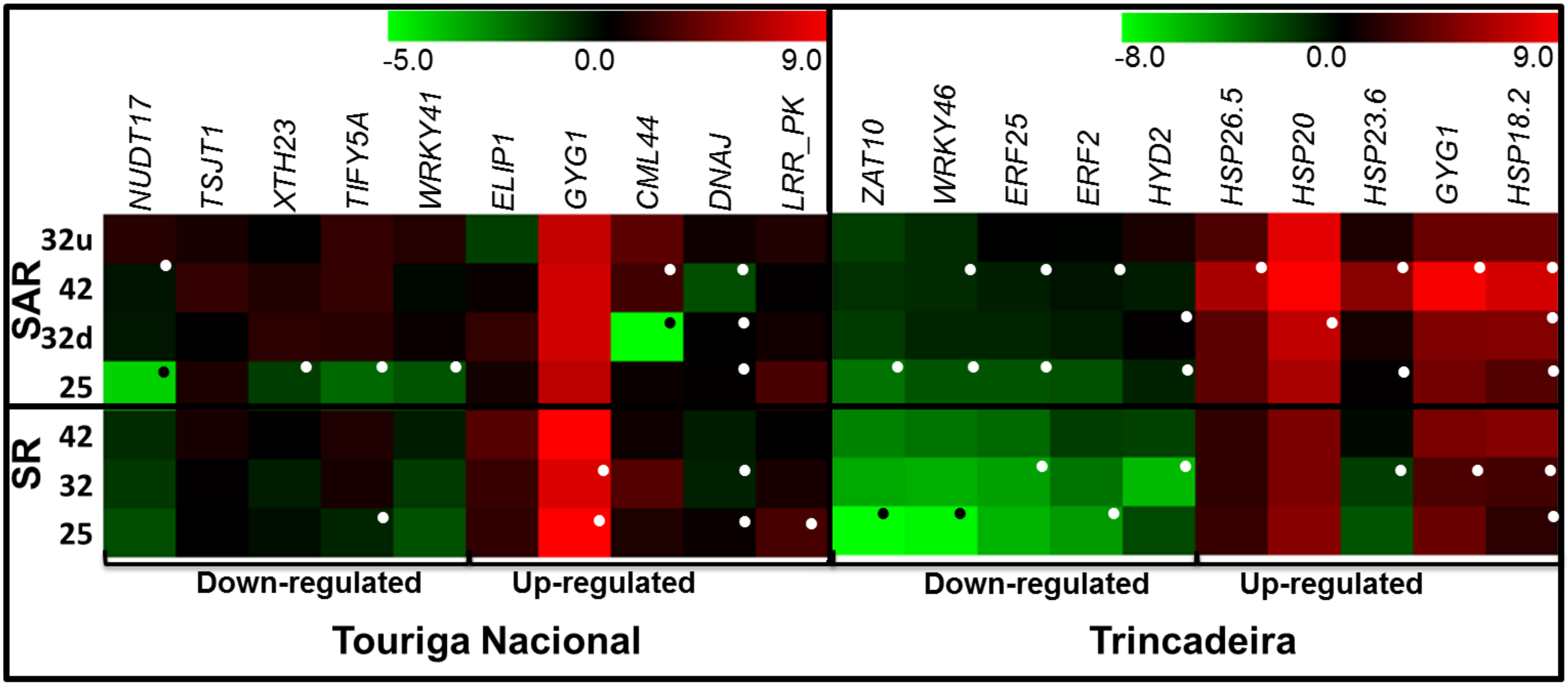

Figure S1. Relative gene expression ratios obtained by RT-qPCR of the five most up-regulated and the five most down-regulated transcripts obtained when the 23K custom-made Affymetrix Vitis GeneChip was applied to TN and TR after acute heat stress (1h at 42ºC) (Rocheta *et al.* in preparation). The transcripts were monitored in both varieties in SAR and SR and are the following: .small heat shock protein 26.5 kDa (*HSP26.5*), small heat shock protein HSP20 (*HSP20*), small heat shock protein 23.6 kDa (*HSP23.6*), glycogenin-1 isoform 1 (*GYG1*) and class I heat shock protein 18.2 kDa (*HSP18.2*) as the five most up-regulated transcripts in TR; zinc finger protein ZAT10-like (*ZAT10*), WRKY transcription factor 46 (*WRKY46*), ethylene responsive factor ERF025 (*ERF025*) ethylene responsive factor ERF2 (*ERF2*) and hypodontia, autosomal recessive 2 (*HYD2*) as the five most down-regulated transcripts in TR; early light-inducible protein 1 (*ELIP1*), *GYG1*, calcium-binding protein CML44 (*CML44*), chaperone protein DnaJ (*DNAJ*) and LRR receptor, serine/threonine-protein kinase At3g47570-like (*LRR_PK*) as the five most up-regulated transcripts in TN; nudix hydrolase 17 (*NUDT17*), stem-specific protein TSJT1 (*TSJT1*), xyloglucan endotransglucosylase/hydrolase XTH23 (*XTH23*), protein TIFY 5A (*TIFY5A*) and WRKY transcription factor 41 (*WRKY41*) as the five most down-regulated transcripts in TN. Relative expressions in relation to control plants in each treatment and stress moment were calculated and values were normalized with respect to translation initiation factor eIF-3 subunit 4 (*TIF*), translation initiation factor eIF-2B alpha subunit (*TIF-GTP*) and actin 2 (*act*) mRNA. The data correspond to $\log_{2(\text{fold-expression})}$ of three independent samples measured in duplicate. Values within $\log_{2(\text{fold-expression})} < | 2 |$ are not significantly different from the controls. Statistically significant differences between a TN/TR stress moment and the previous one after Tukey's multiple comparison tests for a p value lower than 0.05 are indicated by •.
